# Structural Consequences of Copper Binding to the Prion Protein

**DOI:** 10.3390/cells8080770

**Published:** 2019-07-25

**Authors:** Giulia Salzano, Gabriele Giachin, Giuseppe Legname

**Affiliations:** 1 Department of Neuroscience, Scuola Internazionale Superiore di Studi Avanzati (SISSA), 34136 Trieste, Italy; 2 European Synchrotron Radiation Facility (ESRF), 38043 Grenoble, France; 3 ELETTRA Sincrotrone Trieste S.C.p.A, Basovizza, 34149 Trieste, Italy

**Keywords:** prion protein, copper binding, copper coordination geometries, neurodegenerative diseases

## Abstract

Prion, or PrP^Sc^, is the pathological isoform of the cellular prion protein (PrP^C^) and it is the etiological agent of transmissible spongiform encephalopathies (TSE) affecting humans and animal species. The most relevant function of PrP^C^ is its ability to bind copper ions through its flexible N-terminal moiety. This review includes an overview of the structure and function of PrP^C^ with a focus on its ability to bind copper ions. The state-of-the-art of the role of copper in both PrP^C^ physiology and in prion pathogenesis is also discussed. Finally, we describe the structural consequences of copper binding to the PrP^C^ structure.

## 1. Introduction

Prion diseases or transmissible spongiform encephalopathies (TSEs) are fatal neurodegenerative disorders that affect humans and a wide range of mammalian species. These disorders can arise sporadically, be inherited, or be acquired through infection. The sporadic forms are the most common and in humans sporadic prion diseases account for approximately 85% of all cases [1]. They include sporadic Creutzfeldt–Jakob disease (sCJD) [2], sporadic fatal insomnia, and the variably protease-sensitive prionopathies [3]. Genetic forms of prion diseases are associated with mutations in the human prion protein gene (*PRNP*) and comprise familial CJD (fCJD), Gerstmann–Straussler–Scheinker (GSS) syndrome [4], fatal familial insomnia (FFI) [5] and prion protein cerebral amyloid angiopathy [6]. Acquired prion diseases are very rare, occurring in less than 1% of the cases. They are transmitted from human to human, as iatrogenic CJD and Kuru, or from cattle to humans, as variant CJD (vCJD) [7]. Animal TSEs are scrapie in sheep and goats, bovine spongiform encephalopathy (BSE) in cattle and chronic wasting disease (CWD) in deer, elk and moose [8]. BSE spread from the UK to at least 28 other countries, mostly in Europe, with occasional cases also confirmed in Asia and North America, while CWD has slowly spread around 26 states in the United States of America, as well as 3 provinces in Canada and in South Korea [9,10]. Notably, the first European cases of CWD have been recently identified in free-ranging reindeer, moose and red deer in Norway, Finland, and Sweden [11,12,13,14]. Interestingly, a first case of dromedary camel prion disease (CPD) has been recently reported in Algeria [15].

The neuropathological hallmarks of TSEs are spongiosis, glial proliferation, and neuronal loss. They are caused by the misfolding of the physiological cellular prion protein (PrP^C^) into its pathogenic scrapie isoform (PrP^Sc^), an insoluble and partially protease-resistant isoform that is able to propagate by interacting to and converting PrP^C^ into nascent prion molecules [16].

PrP^C^ is a ubiquitous glycosylphosphatidylinositol (GPI)-linked glycoprotein, highly conserved among mammals [17], mainly found in the central and peripheral nervous systems [18,19]. As a typical cell-surface glycoprotein, the pre-pro-protein is synthesized in the endoplasmic reticulum (ER), due to the presence of a 22 amino acids N-terminal signal peptide that is then cleaved into the ER lumen. Here, the immature prion protein is subjected to several post-translational modifications including N-linked glycosylation, formation of a disulfide bond, cleavage of the C-terminal signal peptide and subsequent attachment of the GPI anchor at position 231 [20,21,22]. The mature GPI-anchored form, PrP^C^, is trafficked through the Golgi apparatus where further processing of the N-linked oligosaccharides results in modified glycosylation to complex-type sugar chains, depending on the number of glycosylation sites occupied with oligosaccharide chains [23]. The mature PrP^C^ is found mostly in the cholesterol- and sphingolipid-rich membrane domains, also known as lipid rafts, which are detergent-resistant membrane domains with many important cellular receptors and other GPI-anchored proteins [24].

## 2. The Structure of PrP^C^ and PrP^Sc^

After attachment to the outer leaflet of the plasma membrane via a C-terminal GPI anchor, the mature PrP^C^ consists of 209 residues including up to two N-linked glycans at asparagines 181 and 197 (human numbering). Atomic structures obtained by NMR techniques and X-ray crystallography revealed that PrP^C^ shares a very similar fold across different mammalian species. The globular C-terminal domain of PrP^C^ (residues 128–231) contains three α-helices (α1, α2 and α3) and two short anti-parallel β-sheets (β1 and β2) [25]. Recently, an additional short β-sheet (denoted as β0) has been identified [26]. Helices 2 and 3 are covalently bridged by a single disulfide bond between Cys179 and Cys214, essential in forming and maintaining the tertiary fold. The N-terminal domain of PrP^C^ (from residue 23 to 127) is largely unstructured [27] and it is composed of four different consecutive domains, or octapeptide repeats (OR) (residues 60–91), each carrying histidines and tryptophan able to coordinate up to four copper ions with different coordination geometries and high affinity [28]. Additional histidines (His96 and His111) are able to bind copper, they are located in the “fifth” or non-OR copper binding site (residues 92–111) [29]. The adjacent region is a hydrophobic domain that includes the palindromic motif of sequence AGAAAAGA (residues 113–120), which is thought to play a role during prion conversion [30]. PrP^C^ can also undergo proteolytic processing [31]. The alpha-cleavage is the main proteolytic event of PrP^C^. It is located at His111 and leads to the production of two fragments: the N-terminal fragment, N1 (23–110 residues) and a C-terminal fragment, C1 (111–231) [32].

Unlike PrP^C^, the PrP^Sc^ structure features mostly β-sheet secondary structure motives. These unique structural features of PrP^Sc^ are responsible for its physicochemical properties, including insolubility in non-ionic detergents, partial proteinase-K (PK) resistance, and aggregation propensity [33]. Its insoluble and heterogeneous nature renders its structural characterization extremely difficult. To date several prion structural models have been proposed, two of them seem to be the most accepted: the parallel in-register intermolecular β-sheet (PIRIBS) and the 4-rung β-solenoid models [34,35]. The 4-rung β-solenoid model is in agreement with experimental constraints of brain derived PrP^Sc^ obtained through cryo-EM and X-ray fiber diffraction studies [36,37] and allows accommodation of the bulky glycans present in brain-derived PrP^Sc^ [38]. On the other hand, the infectious PrP23–144 amyloid [39] exhibits a PIRIBS architecture, and solid-state NMR studies revealed structural differences between PrP23-144 amyloid fibrils from different species, providing a structural basis for understanding the species barrier phenomenon [40,41].

## 3. PrP^C^ Functions Suggested by Copper Binding

The most well-known function of PrP^C^ is its ability to bind metal ions through its flexible N-terminal moiety. Since the first observation of PrP^C^ as a copper-binding protein [42], many attempts have been made to understand the role of metal ions in both PrP^C^ physiology as well as in prion pathogenesis. Indeed, the molecular details of PrP^C^ metal binding are now well understood. In contrast, the effects of metal binding on the in vivo function of PrP^C^ are largely unknown, in part due to the complexity of the metal metabolism and because the interaction of proteins with metals is often missed due to low affinity or their transient nature [43]. Nevertheless, to date evidence supports the idea that the physiological function of PrP^C^ is related to its metal-binding properties.

A role for Cu(II)-binding PrP^C^ in stimulating endocytosis and trafficking of PrP^C^, acting as a copper uptake protein has been suggested [44,45,46,47,48]; other investigations propose a role for PrP^C^ as an antioxidant, with a copper-dependent enzyme function with SOD-like activity [49]. PrP^C^ is also involved in neuritogenesis [50] through its N-terminal domain [51]. This may be correlated to its role in memory and cognition synaptic regulation [52].

Other studies proposed PrP^C^ functions attributed to the PrP^C^ ability to bind copper are related to *N*-methyl-d-aspartate (NMDA) receptors modulation [53,54,55] and brain metal homeostasis [56]. Some others point out the role of Cu(II) either as promoter or attenuator of β-sheet conversion and amyloidal aggregation [57,58,59,60].

PrP^C^ binds copper in vivo [61] and cultured murine cells and neurons chronically exposed to high Cu(II) concentrations show increased expression of *Prnp* [62]. It was shown that the addition of copper significantly increased PrP^C^ levels in neuronal cells, while the co-treatment with bathocuprionedisulfate (BCS), a copper chelator, re-established normal PrP^C^ expression levels.

It has also been reported that copper stimulates rapid endocytosis and trafficking of PrP^C^, functioning as a copper uptake protein [44,45,46,47,48]. PrP^C^ is predominantly localized to presynaptic membranes, where copper ions are highly localized [45]. Lower copper concentration in synaptosomal preparations of *Prnp*^0/0^ mice suggest that PrP^C^ plays a role in regulating copper concentration at the synapse of neurons as well as in the re-uptake of the metal into the presynaptic cleft [47]. Furthermore, copper added to cultured neuroblastoma cells promotes the endocytosis of the PrP^C^ [44,46]. Therefore, it can be hypothesized that the transport of copper from the extracellular to the intracellular compartment is operated through PrP^C^ internalization. Alternatively, PrP^C^ may act as a copper buffering molecule at the synaptic cleft, capturing copper and handling it over to other membrane transporters. By using PrP^C^ mutants lacking either the OR region or the N-terminal polybasic region, Taylor et al. shown that copper binding to the OR promotes dissociation of PrP^C^ from lipid rafts towards detergent-soluble regions of the plasma membrane, while the N-terminal polybasic region mediates its internalization by clathrin-mediated endocytosis [63].

An evolutionary relationship between PrP^C^ and ZIP (Zrt-, Irt-like Protein) family of zinc transporters supports the function of PrP^C^ in relation with its metal ion regulation [64]. Indeed, it has been shown that PrP^C^ regulates the amount and distribution of specific metals within the central nervous system, thus regulating the progression of neurodegenerative diseases in which altered metal homeostasis might have a role in Alzheimer’s and Parkinson’s diseases [56]. Pocanschi et al. suggest that ZIP5 was found to have the same subcellular locations as PrP^C^, when expressed in neuroblastoma cell lines [65]. Interestingly, they found that ZIP5 undergoes N-glycosylation within its PrP-like domain, and it may acquire a dimeric, globular fold similar to that of mammalian PrP^C^ [65]. The observed co-localization between PrP^C^ and ZIP5 might indicate that the two proteins have retained, throughout evolution, features responsible for their subcellular localization [65].

Consistently with PrP^C^ localization in the synaptic region together with its high affinity to copper ions, PrP is thought to possess a copper-dependent enzyme function with SOD-like activity, enabling its effective function as an antioxidant in the central nervous system [49]. Mouse PrP^C^ either as recombinant protein or immunoprecipitated from brain tissue has been shown to have the activity of a copper/zinc-dependent superoxide dismutase (SOD), endowing PrP^C^ with antioxidant capacities [66]. In vivo studies show that the activity of cytosolic SOD is reduced in the brains of PrP-knock out (KO) mice [49], while increased in mice that overexpressing PrP^C^ [67]. Moreover, SOD activity in brain lysates from WT mice was reduced after PrP^C^ removal using anti-PrP^C^ antibodies [68]. However, studies with PrP-KO mice show decreased SOD activity, without increased susceptibility to oxidative stress [67], while other studies have shown that PrP does not exhibit SOD-like activity in vivo nor in vitro [69,70]. Although the role of PrP^C^ as a SOD or as an oxidative stress-reducer is not yet definitive, PrP^C^ clearly has a neuroprotective role.

Additional evidence about physiological functions of PrP^C^ arise from studies on deletions in the N-terminal domain showing that PrP^C^ may protect against excitotoxic stress in neurons. While the deletion of the entire N-terminal domain has no clinical symptoms or neuropathology [71], removing 21 residues (105–125) from the N-terminal domain of PrP (PrP ΔCR) induces spontaneous neurodegeneration in transgenic mice, without accumulation of PrP^Sc^ [72,73]. Interestingly, this neurotoxic phenotype can be suppressed by co-expressing WT PrP^C^ [74,75], suggesting that WT and deleted PrP^C^ might interact with each other or by competing for a binding to a specific molecule at the cell surface.

PrP^C^ has been also considered as a neurotrophic factor that facilitates neurite outgrowth and growth cone (GC) guidance through NCAM-Fyn-ERK pathway [76,77]. This process is clearly inhibited if the copper binding site is disrupted with a consequent altered copper coordination [51].

It has also been established that PrP^C^ regulates *N*-methyl-d-aspartate receptor (NMDAR), protecting neurons from glutamate-induced excitotoxicity [54,55,78,79]. Impairments in NMDAR activity induces excitotoxicity in PrP-KO mice causing massive calcium influx thus leading to neuronal cell death pathway. Co-expression of PrP^C^ WT allows PrP^C^ to inhibit the NMDAR, leading to suppress excitotoxicity [54]. This process requires the presence of copper. Interestingly, this protective role of PrP^C^ is consistent with recent studies, in which overexpression of mouse PrP^C^ induces desensitization of NMDA currents, while overexpression of human PrP^C^ had an opposite effect, suggesting that the NMDA receptor could be differentially sensitive to the conformation structure adopted by human and mouse PrP^C^ [80]. Gasperini et al. showed that PrP^C^ exerts copper-dependent neuroprotection by inhibiting NMDAR through S-nitrosylation, a post-translational modification resulting from the reaction of nitric oxide (NO) with cysteines [53]. In particular, they found that NMDAR S-nitrosylation decreased in PrP-KO mice and copper chelation decreases NMDAR S-nitrosylation in WT but not in PrP-KO mice. They proposed a neuroprotective mechanism of PrP^C^, in which PrP^C^-bound copper ions act as electron acceptors in the reaction between NO and NMDAR cysteine thiols. The consequent S-nitrosylation inhibits the NMDAR, leading to a reduction of the neurotoxic effect caused by its overactivation. In a subsequent study, the NMDAR S-nitrosylation levels were analyzed at pre- and post-symptomatic stages of mice intracerebrally inoculated with RML, 139A, and ME7 prion strains [81]. It was found a reduction of the levels of NMDAR S-nitrosylation at both pro- and pre-symptomatic stages, suggesting an impairment of NMDAR S-nitrosylation as a mechanism causing neuronal death in prion diseases. Therefore, the progression of excitotoxicity might be blocked by restoring NMDAR modulation and preventing neuronal loss thus opening the possibility for new therapeutic approaches against prion disorders [81]. These findings underlined the role of Cu-bound to PrP^C^ in neuroprotective mechanisms, which could be altered by the presence of ligand that interfere with copper binding, as the case of amyloid-β (Aβ) that might mediate neuronal and synaptic injury by disrupting the normal NMDAR activity [82].

The PrP metal-binding property has been used to purify recombinant prion protein [83,84] as well as natively folded PrP^C^ isolated from brain tissue [85,86,87,88] by using Cu(II)-loaded immobilized metal affinity chromatography (IMAC) columns, as the PrP copper binding sites work as an affinity tag for such columns, thus enabling one-step purification of proteins without the use of histidine tag [83]. Interestingly, the ability of PrP^C^ to bind Cu(II)-loaded IMAC columns is strongly dependent on the glycosylation state of the protein [89]. The non-glycosylated form of the full-length PrP has the highest binding affinity for metal ions among the different PrP glycoforms. The fact that either one of the two glycans are sufficient to alter the metal ion-binding capacity of PrP might be correlated to the presence of interactions between the C-terminal located glycans and the N-terminal domain.

Although it was proposed that PrP^Sc^ was not able to bind copper immobilized on Cu(II)-loaded IMAC [87,88], Dron and colleagues have shown that the ability of PrP^Sc^ to bind copper ions has allowed the biochemical characterization of different truncated forms of PrP^Sc^, whose binding affinity dramatically depended on which cell or tissue supported prion replication [90]. Furthermore, it was shown that copper enhances the recovery of both PrP infectivity and PK resistance in samples that did not renature by diluting guanidine hydrochloride, probably by stabilizing the formation of initial aggregates. It might well be that copper promotes the formation of defined structures in PrP monomers and modulates interactions between PrP monomers [91,92].

All PrP^C^ functions involving Cu(II) binding are listed in Table 1.

## 4. Copper-Binding Models

**Figure 1:**
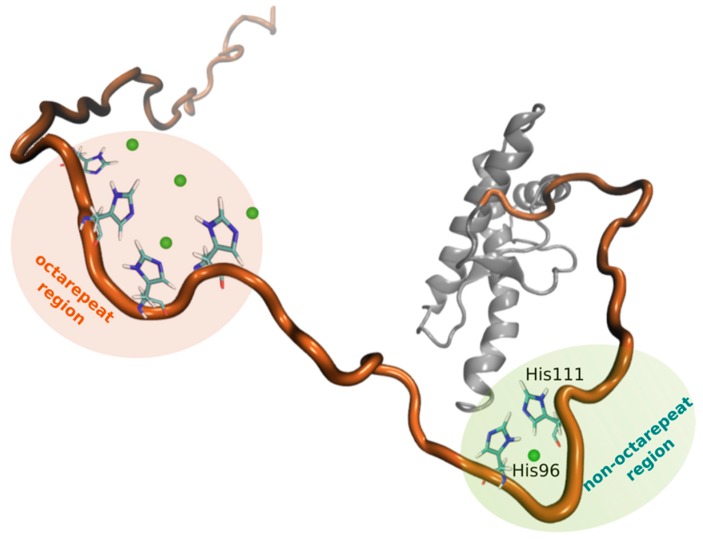
Cartoon representation of the secondary PrP^C^ structure. The globular domain is shown in grey, the N-terminal region in orange. The octarepeat and non-octarepeat regions are highlighted in orange and green, respectively, with histidine residues binding copper ions (in green).

The first observation of PrP^C^ as copper-binding protein has been done by Hornshaw and collaborators [42]. Thenceforth, elucidating the molecular details of copper binding has become of utmost importance for better understanding the role of the copper in PrP^C^ physiology and in the prion pathogenesis. PrP^C^ primarily binds copper through the OR region composed of multiple tandem repeats of the eight-residue sequence PHGGGWGQ (Figure 1). Across species, the OR domain is the most highly conserved regions of PrP^C^ sequence, even if some species carry four or five OR repeats [94]. Although X-ray crystallography and solution-state NMR are the canonical approaches for structural determinations, each of them encounters technical problems due to the presence of copper. For example, the presence of paramagnetic Cu(II) in NMR leads to significant line broadening [95]. To overcome these problems, a wide range of electron paramagnetic resonance (EPR) methodologies have been explored to study the structural features and affinities of the Cu(II) to the OR [96]. By using EPR analysis of a series of octarepeat peptides, it was shown that the octarepeat domain takes up four equivalents of copper, in which the coordination environment is composed of three nitrogen atoms and one oxygen atom. The residues HGGGW in each repeat represent the fundamental Cu(II)-binding unit [97]. Then, by using a library of site specifically ^15^N-labeled octarepeat peptides, it was shown that the two glycine (Gly) residues following the His coordinate through their amide nitrogens, while the ^14^N of the third Gly is not coordinated to the Cu(II) center, being 4.0 Å away. These EPR experiments are in agreement with the X-ray crystal structure of the Cu(II)-HGGGW complex [98] which indicate a square pyramidal geometry, as well as with subsequent computational [99] and NMR studies [100]. The EPR methodologies shown that the molecular features of copper coordination in the octarepeat domain depend on the amount of copper bound [101]. Three different binding components at pH 7.4 were identified. *Component 3* represents the low-occupancy mode of interaction with multiple His residues coordinated a single Cu(II) with high affinity (as indicated by the dissociation constant (K_d_) of 0.1 nM). In contrast, higher copper occupancy -corresponding to the *component 1* mode- involves coordination through deprotonated amide nitrogens and exhibits a weaker affinity with a K_d_ of 10 μM. The intermediate state is represented in the *component 2*, where each Cu(II) coordinates two His residues, thus forming intervening loops. It has been suggested that the OR domain does not play a role in TSEs, since treatment with protease-K (PK) removes the OR regions without loss of infectivity [102]. Moreover, it was shown that addition of copper to PrP^C^ converts the protein to a partially PK-resistant state, and this conversion requires only a single Cu binding site [103].

Studies by Qin’s [104] and Millhauser’s groups [97] showed that another Cu binding takes place outside the OR region, called non-OR or fifth copper-binding site, involving His96, which is adjacent to the octarepeats and, like the octarepeats, is in a glycine rich environment (Figure 1). By using circular dichroism to detect copper binding in peptides including His111, Jones et al. [57] showed that both His96 and His111 bind copper. In particular, they found three coordination modes there were strongly influenced by pH. All of them display a square planar geometry. At pH 7.5 and above, a 4N complex dominates, while at pH 6.0, a ligand rearrangement shifts the coordination to a 3N1O configuration. At low pH, a multi-histidine residue 2N2O coordination dominates. In line with this, it was concluded that His96 and His111 bind copper independently, except at lower pH where both His residues coordinate a single copper atom. The involvement of both His96 and His111 has also been confirmed by NMR studies [59,105] as well as by analysis of metal catalyzed oxidation [106]. Data suggest that His96 is the key site involved in copper binding [107]. Importantly, it was shown the existence of a cooperative effect between Cu(II) binding to the N-terminus and to the non-OR copper binding site that is mainly provided by His96 [107]. However, a recent spectroscopic study suggested that the His111 has a slightly higher affinity for Cu(II) than His96, with Met109 playing a key role in the binding preference of Cu(II) for the His111 site [108].

The role of copper binding to the OR and non-OR regions was also investigated by a combination of genomic and cell biological approaches, and a focal stimulation technique. Here, by substituting histidine residues with tyrosine located in the OR and non-OR region, alteration of the copper-binding sites perturbed the PrP^C^ structural conformation, thus failing to induce neurite outgrowth signal [51].

By using different spectroscopic techniques, studies have also focused on the Cu(II) coordination to the C1 fragment, which includes the His111, as result of the neuroprotective alpha-cleavage [109]. By using the PrP(111–115) fragment as a model system, it was shown that the His111 and the free NH_2_ group act as an anchoring site for Cu^2+^, resulting in different coordination modes, depending on proton and copper concentrations ([109] and reviewed in [110]).

Interestingly, several studies suggest another copper-binding site located in the C-terminal domain of PrP^C^. By using a series of His/Ser mutants of the C-terminal His residues, only H140S mutant remained folded in the presence of copper, but no relevant changes in copper binding were observed. The same behavior was observed for H177S mutant for which no change in copper binding was shown. Therefore, the authors proposed H187 as putative copper binding site [111,112,113]. Interestingly, H187 is the site of a pathogenic mutation causing GSS-like disease in humans [114]. However, it seems that copper binding in the C-terminal domain occurs only at very high copper levels, which is unlikely to be physiologically relevant. This suggest a predominant, high-affinity copper coordination to PrP^C^ restricted to the N-terminal domain of PrP^C^ [97].

## 5. Copper and Prion Diseases

A connection between prion disease and copper metabolism was first proposed in the early 1970s. Pattison and Jebbett [115] showed that a copper chelator, cuprizone, induces spongiform encephalopathy and gliosis, similar to the histopathology observed in mouse scrapie. The work of Kimberlin and Millson also found that cuprizone could delay (with very low inoculation titers) the terminal illness in mice affected by scrapie [116]. After those studies, the issue seems to have been largely overlooked for about two decades. The relationship between copper and prion diseases came back into hot debate in 1997 when copper was found to bind PrP^C^ [117]. There are still controversial conclusions about the deleterious or beneficial effects of copper in prion diseases. Several studies strongly support a beneficial role of copper against prion disease progression. It was showed that supplementing copper in the diet prolonged the survival time in infected animals [118,119]. Moreover, N2a cells treated with copper do not bind and internalize PrP^Sc^ [119]. Other groups suggest that copper treatment interferes with the propagation of PrP^Sc^ in ScN2a cell lines while there is an accumulation of total PrP^C^ [120]. Instead, Sigurdsson and colleagues found that treatment of scrapie-infected mice with a copper chelator delayed the onset of prion disease, suggesting that copper has a prion promoting effect [121]. Additionally, copper has been found to convert PrP^C^ into protease-resistant and detergent-insoluble forms [103]. Fluorescence experiments show that Cu(II) promotes PK resistance through direct interaction with PrP(23–231) and not by inhibiting PK itself as the truncated PrP(90–231) exposed to Cu(II) remains more PK-sensitive. Moreover, only the full-length PrP exposed to Cu(II) ions act as seed for the formation of PrP aggregates in vitro in the RT-QuIC seeding assay [122]. The effect of Cu(II) binding on the fibrillization reaction has been also investigated recently by Qi et al. They found that fibrillization does not abolish the ability of PrP to bind Cu(II) in both monomeric and fibrillar forms. Furthermore, it seems that fibrillar form of PrP is no longer able to bind copper at the His96, suggesting that somehow this region becomes inaccessible in the fibrillar state [123].

Consistently, a study on PrP expressed in yeast showed that supplement of copper in the yeast growth media leads to the formation of PK-resistant forms [124]. A misfolding cyclic amplification (PMCA) study showed that PrP^Sc^ in the presence of copper could propagate and form more prions in the presence of deoxycholic acid [125]. Similar but contrasting results come from a study showing that copper inhibits PrP^C^ to PrP^Sc^ conversion in PMCA [126] as well as formation of fibrils from recPrP in an amyloid seeding assay [120]. The latter study is in line with another RT-QuIC assay in which the inhibition of fibril formation of recombinant elk PrP seems to be correlated to the presence of copper ions in the reaction [127].

Wadsworth and colleagues have investigated the metal-ion occupancy in PrP^Sc^ isoform isolated from two biochemically distinct strains of sporadic CJD [128]. By using metal-chelating agents to disrupt PrP^Sc^ copper and zinc binding sites, they found an altered electrophoretic mobility of the cleavage products after PK digestion in two of the four defined strains of CJD. Therefore, the authors suggested that the ability of metal ions to influence PrP^Sc^ conformation could have implications for understanding molecular mechanism for strain variation [128].

In vivo studies suggest that the absence of the octapeptide repeats still sustains scrapie infection, but with longer incubation times without histopathological signs of scrapie [129]. Moreover, it was shown that PrP fragments with larger deletions that included both OR and non-OR regions caused spontaneous ataxia and degeneration of the granular layer of the cerebellum, highlighting the role of copper binding sites in prion conversion [130]. In contrast, a previous study revealed that the deletion of an octarepeat did not lead to disease [131]. Insertion of nine extra octapeptide repeats in the *Prnp* gene is associated with prion diseases as well [132]. Interestingly, Tg(PG14) mice expressing nine-octapeptide insertion in PrP, thus resembling the human familial prion disease, spontaneously developed a fatal neurodegenerative disorder, but they were characterized by the accumulation in the brain of weakly protease-resistant form of PrP mutant which was not infectious in animal transmission experiments [133].

The studies discussed above clearly show that there is a debate about the role of the copper in facilitating or inhibiting prion formation. Additional studies are required to further investigate the relationship between copper and prion diseases.

## 6. Structural Consequences of Copper Binding

Although the PrP^C^ N-terminal and C-terminal moieties have often been described as independent domains, several studies now suggest that copper ions promote specific N- to C-terminal interactions. A recent study showed that copper bound to OR and non-OR regions induce major changes in the interdomain conformation of the protein and proposed that the non-OR region acts as anchor of the two halves of the protein [134]. Previously, Thakur et al. reported novel long-range inter-domain interactions of the N- and C- terminal regions of full length recPrP upon Cu(II)-binding, resulting in significant compactness of recPrP structure upon Cu(II)-binding. In particular, they found that the region 90–120, containing the binding site His96 and His111, becomes proximal to the α1-helix for interaction upon copper binding [58]. Subsequent EPR and NMR studies showed that Cu(II)-binding to the OR region alters residues located nearby the β1-α1 loop and the α2-α3 loop region [135,136]. Recently, the same authors have identified the residues-level contacts between the N-terminal polybasic domain (including the segment 23-31 and the non-OR region) and a C-terminal negatively charged epitope coincident with the α1-β2-α2 loop region as well as the end of α3, detailing how the contacts are altered by binding of Cu(II) [134].

The influence of copper ions on structural rearrangement of octarepeat regions from human and chicken PrP has been also investigated [137]. In the presence of sodium dodecyl sulfate (or SDS) to mimic the membrane environment in vitro, authors observed different copper coordination modes in both human and chicken OR sequences. While three histidines are involved in binding for the human OR, the binding involves four histidines for the chicken OR, suggesting that the aromatic ring of tyrosine residues present in the chicken OR sequence may stabilize copper anchoring site [137]. Interestingly, both human and chicken OR regions were found to bind copper ions more efficiently than the corresponding amyloidogenic fragments, probably due to the presence of four histidines compared to the two histidines in the amyloidogenic sequence [138].

**Figure 2:**
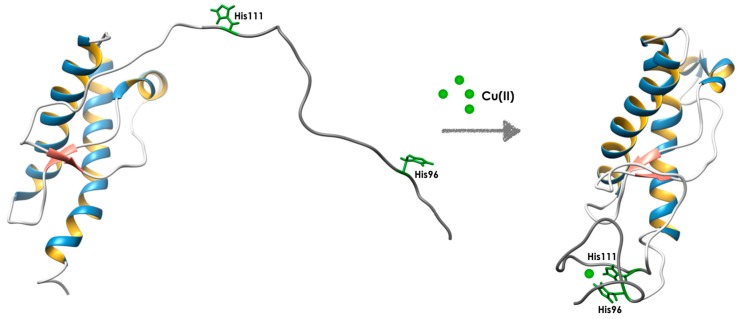
Cartoon representation of the structural rearrangement of PrP^C^ structure induced by Cu(II)-binding to the non-OR region involving the His96 and His111 residues [140].

It has also been studied the effect of the pathological point mutations on copper coordination when the metal is bound only to the non-OR region. By means of X-ray absorption spectroscopy, D’Angelo et al. highlighted a significant modification of the non-OR Cu(II) binding site caused by the pathological point mutation Q212P, located in the globular domain, reinforcing the hypothesis that the non-OR copper binding site is strongly influenced by the interactions between the C- and N-terminal domains [139]. The involvement of H96 and H111 in non-OR region shows that Cu(II) occupancy plays a role in determining the conformation of PrP^C^. It was observed an alteration of Cu(II) coordination due to the presence of a mutation that abrogates a copper ligand, H96Y, and causes spontaneous PrP^Sc^-like formation in neuronal cultured cells and accumulation in the acid compartments [140]. The authors proposed a model whereby HuPrP coordinating copper with His96 and His111 in the non-OR region is more resistant to prion conversion compared to the protein coordinating Cu(II) with one histidine [140] (Figure 2). Molecular dynamic simulations also revealed alterations of hydrogen bond network in PrP^C^ coordinating one His, thus creating favorable conditions for transient β-sheet motif formations [140]. The effect of Cu(II)-binding was also assessed on the oligomerization of PrP^C^. Lin et al. showed that Cu(II)-binding promoted oligomerization of a susceptible species more significantly than that of a resistant species, suggesting that the low susceptibility to Cu(II) in the resistant species might results in a weak risk of Cu(II)-induced TSE diseases [141]. In support to the role of the non-OR region for prion conversion, transgenic mice, TgPrP(H95G), with an amino acid replacement at residue H95 showed shorter disease progression than WT control mice and classical clinical signs of TSE [142]. Evidence about the role of copper ions in mediating structural changes of the N-terminal domain of PrP^C^ come from a work by Lu et al. By using the PrP(23-89) peptide, it was shown that Cu(II) induces the formation of PK-resistant material detected by western blot and atomic force spectroscopy (AFM), and structural changes detected by hydrogen/deuterium exchange in the N-terminus of PrP^C^ [143].

## 7. Conclusions

The link between PrP^C^ and copper is likely to elucidate PrP^C^ function, but also to provide important new insights into the molecular basis of prion diseases. The N-terminal PrP^C^ domain binds copper ions through the OR and non-OR domains and this binding yields a diverse range of Cu(II) coordination modes, each with a distinct binding affinity and geometry [28,139,140]. In this review we have highlighted how copper displays site-specific effects on PrP^C^ folding either promoting stabilizing interactions [58] or inducing local conversion to beta-sheet folds [140]. While the atomic details of Cu(II)-mediated structural changes remain to be fully understood, convincing evidence indicates that copper mediates stabilizing interactions between the N-terminal and the C-terminal domains leading to a compact PrP^C^ folding [134]. Although the functional implications of this Cu(II)-mediated structural change remain to be investigated, this interdomain interaction may play an important role in the physiological activity of PrP^C^ consistent with a proposed role for the protein as transporter, sensor of divalent metal ions and NMDA receptor modulator [53,55,144].

The role of copper in TSE needs to be defined by conciliating both in vitro and in vivo observations. Cu(II)-induced changes of PrP^C^ folding observed in vitro and experiments in animal models suggest a role of copper as a prion-promoting element; on the other hands experiments in cell and animal models report the opposite. As mentioned, treatments of scrapie infected mice with a copper chelator delayed the TSE onset and reduced copper levels in brain and blood, thus supporting the notion that copper exerts a prion promoting effect [121]. An opposite result showed that copper administration to scrapie-infected hamsters delayed the onset of prion disease [119]. In support to the proposed beneficial role of copper against prion disease progression, different studies reported that copper treatment in scrapie-infected N2a cells interferes with prion propagation [145] while the addition of cuprizone promoted a significantly increase of prion PK resistance levels, suggesting that PrP^C^ in the *apo* form is more susceptible to PrP^Sc^ conversion [140].

The protective versus prion promoting effect of copper could depend on an array of variables that are hard to untangle, which include, for instance, the status of the cholesterol metabolism that influence the Cu(II)-mediated PrP^C^ endocytosis [63,146,147,148], the different copper chelators used to treat both animal and cellular models (e.g., cuprizone versus d-penicillamine [116,121]), the homeostasis of copper and other biometals binding PrP^C^, such as zinc and iron, during neurodegeneration [149,150,151,152] and finally the different animal and cell culture models for prion diseases used to understand the role of copper in disease progression.

Copper homeostasis is essential for normal physiology, as highlighted by the spectrum of diseases caused by disruption of the copper transporting enzymes (e.g., Ctr1, and Atp7a) [153]. Notably, a first Atp7a-mediated copper homeostasis link with prion diseases has been recently proposed. Authors isolated by cross breading a mouse line (Atp7a^brown^) carrying a natural mutation in the Atp7a gene (I483T) which is not linked to lethality but with reduced copper content in the brain. Interestingly, this mutation significantly increased the incubation period of intracranial Rocky Mountain Laboratory (RML) scrapie strain infection [154], supporting earlier reports that copper chelation can delay the onset of TSE [121]. Nevertheless, the amount of neuronal loss and astrocytosis was similar in both wild-type and Atp7a^brown^ groups, indicating that even in the presence of reduced copper concentration in the brain PrP^Sc^ remains capable of causing neurodegeneration and death [154].

A novel investigative approach could be the use of alternative animal models to dissect the role of copper in prion diseases as naturally occurring rodent models for Menkes and Wilson diseases [155], which may also provide new insights into the pathogenic changes related to copper dyshomeostasis in TSE and other neurological disorders.

## Figures and Tables

**Table 1:** PrP^C^ functions suggested by Cu(II) binding.

Cu(II)-Mediated Function	Experimental System	Reference
Endocytosis and trafficking	Cell culture, mice	[44,45,46,47,48,63]
Superoxide dismutase-like activity	Cell culture	[49]
Neuritogenesis	Primary hippocampal cultures	[51]
*N*-methyl-d-aspartate (NMDA) receptors modulation	Organotypic hippocampal culture, primary cell culture, mice	[53,54,55,80,81,82]
Brain metal homeostasis	Cell culture, mice	[56]
Inducing or inhibiting β-sheet conversion and amyloidal aggregation	Recombinant mouse prion protein, recombinant human prion protein	[57,58,59,60]
Increasing expression of *Prnp*	Cell culture, primary cell culture	[62]
One-step purification by using Cu-loaded IMAC column	Recombinant prion protein, brain tissues	[85,86,87,93]
Enhanced reversibility of scrapie inactivation	Mice	[91]
